# Ipecacuanha in Dysentery

**Published:** 1871-02

**Authors:** 


In the *Canada Medical Journal* for October, four cases of acute dysentery are reported, which were treated with large doses of ipecacuanha. They occurred in hospital, under the care of Dr. MacCallum. In every case the success of the treatment was most marked and flattering.

Twenty grains was the maximum dose given, and it always followed a dose of tinct. opii m. xv., given one hour before, and with express instructions that no more than a teaspoonful of fluid was to be given at a time; by this means the tendency to vomit was lessened.

